# Microbial Guardians or Foes? Metagenomics Reveal Association of Gut Microbiota in Intestinal Toxicity Caused by DON in Mice

**DOI:** 10.3390/ijms26041712

**Published:** 2025-02-17

**Authors:** Yujing Cui, Haoyue Guan, Samuel Kumi Okyere, Zixuan Hua, Youtian Deng, Huidan Deng, Zhihua Ren, Junliang Deng

**Affiliations:** 1 Key Laboratory of Animal Disease and Human Health of Sichuan Province, Sichuan Agricultual University, Chengdu 611130, China; yjchoi@163.com (Y.C.); guanhaoyue08@163.com (H.G.); samuel20okyere@gmail.com (S.K.O.); 13708235219@163.com (Z.H.); idyt@outlook.com (Y.D.); denghuidan@sicau.edu.cn (H.D.); 2 Department of Pharmaceutical Sciences, School of Medicine, Wayne State University, Detroit, MI 48201, USA; 3 College of Veterinary Medicine, Henan Agricultural University, Zhengzhou 450046, China; 4 Ministry of Education Key Laboratory for Animal Pathogens and Biosafety, Zhengzhou 450046, China; 5 International Joint Research Center of National Animal Immunology, Henan Agricultural University, Zhengzhou 450046, China

**Keywords:** deoxynivalenol, gut, inflammation, microorganism, metagenome

## Abstract

The role of gut microbiota has become a research hotspot in recent years; however, whether the gut microbiota are involved in the alleviation or exacerbation of Deoxynivalenol (DON) toxicity has not been fully studied. Therefore, the objective of this study was to investigate whether the gut microbiota are involved in reducing or aggravating the intestinal damage induced by DON in mice. Mice that received or did not receive antibiotic-induced intestinal flora clearance were orally given DON (5 mg kg/bw/day) for 14 days. At the end of the experiment, serum, intestinal tissue samples and colon contents were collected for further analysis. DON caused development of severe histopathological damage, such as necrosis and inflammation of the jejunum and colon in mice without gut microbiota clearance. The levels of tight junction proteins ZO-1 and occludin were reduced in the jejunum and colon of mice without gut microbiota clearance. In addition, the mRNA and protein levels of pro-inflammatory cytokines (IL-1β, IL-6, and TNF-α) were increased in mice without gut microbiota clearance. The presence of microbiota exacerbate the intestinal damage induced by DON via changes in gut microbiota abundance and production of gut damaging metabolites.

## 1. Introduction

Gut microbiota plays an important function in maintaining the integrity of the intestinal barrier [1,2] A stable and healthy gut microbiota is characterized by the rich diversity of gut bacteria [3]. Microbiota dysbiosis has been associated with the pathogenesis and development of many intestinal diseases. Recent studies have revealed a positive interaction between the gut microbiota and mycotoxins [4]. Mycotoxins can alter the gut microbiota and cause gut and host health disorders [5]. In addition, gut microbiota can biotransform mycotoxins into bioactive or toxic metabolites which may be harmful to the intestines [6], as many mycotoxin metabolizing microbial strains have been identified from the gut [5]. Even though previous studies have revealed the negative effect of mycotoxins on gut microbiota composition, there is still a dearth of knowledge on how the gut microbiota activity on mycotoxins plays a role in damaging of organs in the body after their exposure. To understand this, we first investigated whether the gut microbiota truly play a role in mycotoxin activity using mice with/without gut microbiota clearance exposed to Deoxynivalenol (DON).

DON is the most common mycotoxin produced by *Fusarium* spp and is mainly found in grains and food crops [7]. DON is reported to cause serious human disorders worldwide, with clinical symptoms including nausea, vomiting, abdominal pain, diarrhea, headache, and fever [8]. In addition, both in vitro and in vivo studies have revealed that DON causes damage to the morphology of the intestine and alters its function [9,10,11]. Several studies have reported that DON exposure causes changes in the composition and relative abundance of gut microbiota. Swine that consumed feed contaminated with DON for 4 weeks showed changes in the population of fecal aerobic mesophilic bacteria and anaerobic sulfite-reducing bacteria [6]. Another study also observed an increase in the population of *Bacteroides*/*Prevotella* and a decrease in *Escherichia coli* population in rats after administering DON for 4 weeks [12].

The present study was performed to test the hypothesis that DON intestinal damage is dependent on the presence of gut microbiota. The results from this study will provide us with basic knowledge for further studies into the exact molecular mechanism and biology involved in gut microbiota aggravation of intestinal damage induced by DON, as well as give us novel therapeutic strategies to prevent or treat mycotoxin toxicity via manipulating the gut microbiota.

## 2. Results

### 2.1. Effects of DON and Antibiotic-Induced Microbiota Depletion on Clinical Observation, Changes in Body Weight and Food Intake of Mice

**Figure 1:**
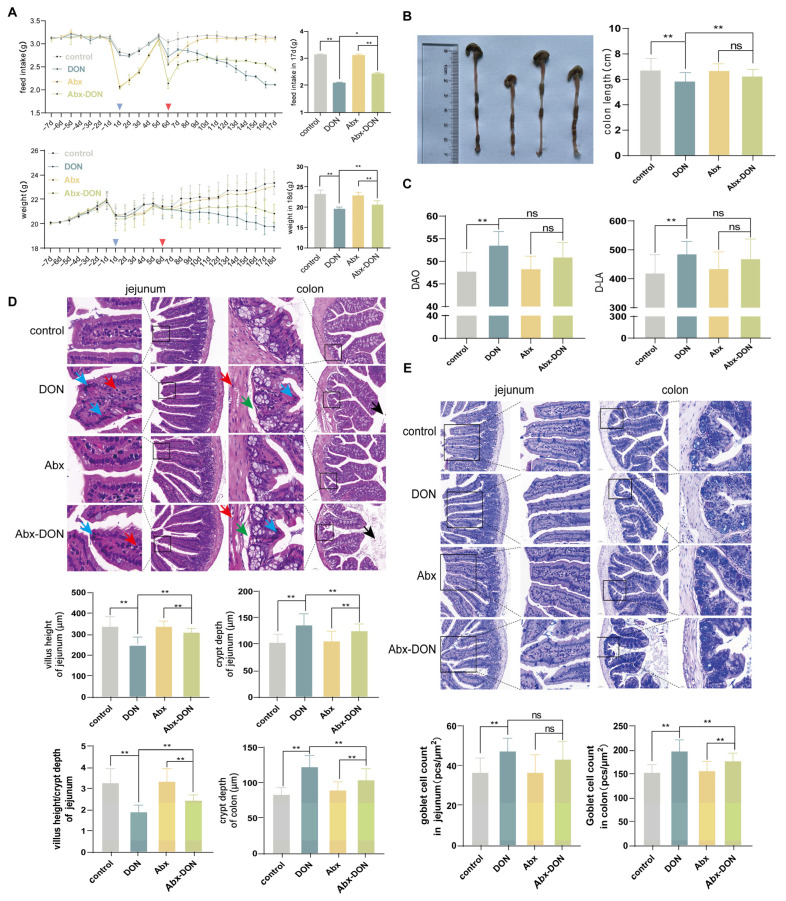
Effects of DON on body weight, feed intake and intestinal pathological injury in mice; (**A**) The effect of DON on body weight in mice during the experiment; the effect of (**A**) DON on feed intake in mice during the experiment, with blue triangles representing the first gavage of quadruple antibiotics and red triangles representing the first gavage of DON; the feed intake of mice in the experiment for 17 days; the weight of mice at 18 days of experiment; (**B**) The effect of DON on colon length in mice. (**C**) Effects of DON and antibiotic-induced microbiome depletion DAO and D-LA; (**D**) H. E staining and Morphological measurement. From left to right, they are 40× (Bar = 20 μm), 20× (Bar = 50 μm), 40× (Bar = 20 μm), 20× (Bar = 50 μm) (Blue arrow: epithelial cell necrosis; red arrow: inflammatory cells present; green arrow: mild edema in the submucosal layer; black arrow: epithelial exfoliated cells in the intestinal lumen) (height of villi in the jejunum, depth of crypts in the jejunum, intestinal villus height/crypt depth and crypts in the colon) (**E**) AB-PAS staining and GCs counting. From left to right, they are 20× (Bar = 100 μm), 40× (Bar = 50 μm), 20× (Bar = 100 μm), 40× (Bar = 50 μm). * *p* < 0.05, ** *p* < 0.01.

The effects of DON on the body weight and feed intake of mice are shown in Figure 1A. After the experimental period, soft stool and lethargy were found in mice administered with DON. We observed that DON exposure significantly lowered food intake and body weight in the DON group compared to the control, Abx and Abx-DON groups (*p* < 0.05). Moreover, the feed intake and body weights of the mice in the Abx group was higher compared to the Abx-DON group (*p* < 0.05) after the experiment.

### 2.2. Effects of DON and Antibiotic-Induced Microbiota Depletion on Colon Length

As Figure 1B shows, we observed that, compared with the control, in Abx and Abx-DON groups, the colon length of the DON group was significantly shortened (*p* < 0.01). However, there was no difference in the colon length between the Abx and the Abx-DON groups (*p* > 0.05).

### 2.3. Effects of DON and Antibiotic-Induced Microbiota Depletion on DAO and D-LA

To further investigate the damage of DON to the intestinal integrity of mice, we measured the levels of DAO and D-LA in mouse serum to detect changes in intestinal permeability. The results showed that, compared with the control group, the DAO and D-LA levels in the serum of the DON group were significantly increased (*p* < 0.01). Similarly, the DAO and D-LA levels in the Abx DON group were increased compared with the Abx group (*p* < 0.01); however, there was no difference in the levels of DAO and D-LA between the DON and Abx DON groups (*p* > 0.05) (Figure 1C). The results showed that DAO and D-LA were only affected by DON, and not by microbiome depletion or combination of the two factors (*p* > 0.05), indicating that DON can damage the intestinal structural barrier by directly/independently increasing intestinal permeability.

The severity of the lesions in each group from mild to severe was as follows: control group ≈ Abx group < Abx-DON group < DON group.

### 2.4. Effects of DON and Antibiotic-Induced Microbiota Depletion on Histological Damage

We observed that the mucosal layer, submucosal layer, muscular layer and outer membrane of each group of jejunum and colon were intact in the control, Abx and Abx-DON groups. We also observed that the lamina propria and the epithelial layer of both the jejunum and colon were clearly demarcated in the control, Abx and Abx-DON groups (Figure 1D). However, administration of DON only caused pathological changes that were mainly characterized by necrosis of epithelial cells in the intestine. Most of the changes occurred in the jejunum at the top of the intestinal villi and in the crypts of the colon. In addition, we observed deepening of the crypts and the appearance of a small number of inflammatory cells in the muscularis, mucosa, submucosa and lamina propria in the DON group. The DON group lost their normal morphology with deepened nuclear and cytoplasm (blue arrows) staining, whereas the epithelial cells showed elongated dark staining, indicating that some epithelial cells were necrotic. We also observed a small number of inflammatory cells in the lamina propria, submucosa and muscularis (red arrows).

We further measured the villi height and crypt depth in the jejunum. The results showed that the crypt depth in the DON group was significantly deeper than that in the control and the Abx-DON groups (*p* < 0.01). In addition, the villus height in the DON group was significantly lower than that in the control group (*p* < 0.01), and the villus height in the Abx-DON group was significantly lower than that in the Abx group (*p* < 0.01). The results from the quantification of the ratio of jejunal villus height/crypt depth showed a significantly lower villi height/crypt depth ratio in the DON group compared to the control group (*p* < 0.01). The villi height/crypt depth ratio of the Abx-DON group was significantly lower than the Abx group (*p* < 0.01), and that of the DON group was significantly lower than the Abx-DON group (*p* < 0.01). The depth of the colon crypts in the DON group was significantly deeper than that of the control and Abx-DON groups (*p* < 0.01). Similarly, the depth of the colon crypts in the Abx group was significantly deeper than that in the Abx-DON group (*p* < 0.01).

### 2.5. Effects of DON and Antibiotic-Induced Microbiota Depletion on Goblet Counting

As shown in Figure 1E, the GCs in the jejunum were distributed within the epithelial layer, while those in the colon were distributed within the developed intestinal glands. The number of GCs in the DON group of the jejunum was significantly increased compared to the control group (*p* < 0.01); however, even though there was an increase in GCs in the Abx DON compared to the Abx groups and DON compared to the Abx DON groups, there was no statistical difference (*p* > 0.05). In the colon, the number of GCs in the DON group were significantly increased compared to the control and Abx DON groups (*p* < 0.01), and similarly, the number of GCs in the Abx DON group was significantly increased compared to the Abx group (*p* < 0.01).

### 2.6. Effect of DON and Antibiotic-Induced Microbiota Depletion on Tight Junction Proteins

**Figure 2:**
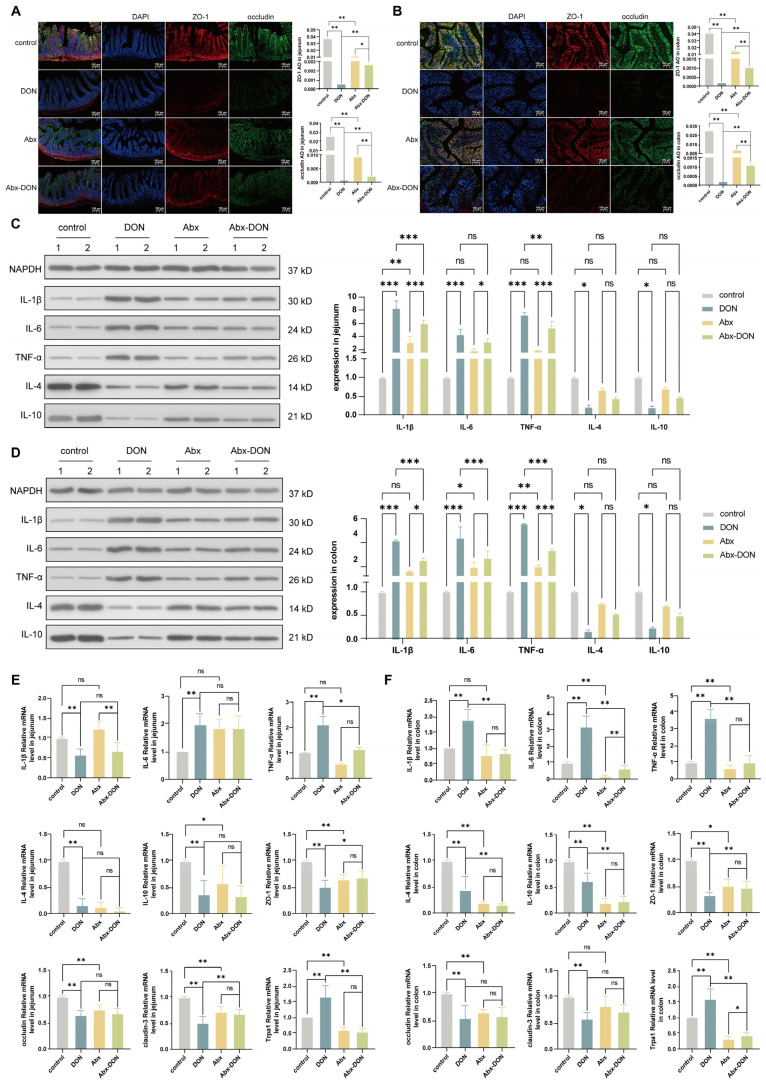
Effects of DON on the mechanical and immune barrier of the intestine. (**A**) Immunofluorescence detection of jejunum tight junction protein (ZO-1 + occludin); (**B**) Immunofluorescence detection of colon tight junction protein (ZO-1 + occludin); (**C**) Western blot test of jejunal inflammatory factor; (**D**) Western blot test for colonic inflammatory factors; (**E**) Jejunum tight junction and inflammatory factor mRNA levels; (**F**) Colonic tight junction and inflammatory factor mRNA levels (n = 8). * 0.01 < *p* ≤ 0.05, ** 0.001 < *p* ≤ 0.01, *** *p* ≤ 0.001.

To investigate the effect of DON and microbiota on the gut integrity, we measured two tight junction proteins (ZO-1 and occludin) in the intestinal tissues. From the results in Figure 2A,B, we observed that the levels of tight junction proteins ZO-1 and occludin in the DON group were significantly reduced compared to the control and the Abx DON groups (*p* < 0.05) in both the jejunum and the colon. In addition, the tight junction protein levels in the Abx group was significantly higher compared to the Abx DON groups (*p* < 0.05). Furthermore, the mRNA expression levels of ZO-1 showed similar results as the protein expression results in both the jejunum and colon; however, there was no significant difference in the ZO-1 mRNA expression levels between the Abx and Abx DON groups (*p* > 0.05). There was no significant difference in occludin mRNA expression levels between the DON and Abx DON and the Abx and Abx DON groups, respectively, in either the jejunum or the colon.

### 2.7. Effects of DON and Antibiotic-Induced Microbiota Depletion on the Protein Expression Level of Inflammatory Cytokines Protein

From the cytokines protein expression results, it was observed that the protein levels of pro-inflammatory cytokines (IL-1β, IL-6 and TNFα) in the DON groups were elevated compared to the control group. In addition, we observed an increased in the levels of IL-1β, IL-6 and TNF-α in the DON group compared to the Abx DON group (*p* < 0.05) in both the colon and jejunum; however, there was no difference in the levels of IL-6 between the DON and Abx DON in the jejunum (*p* > 0.05 or *p* > 0.01). In contrast, the protein expression of anti-inflammatory cytokines (IL-4 and IL-10) showed no significant difference among the groups except between the DON group and control group (*p* < 0.01).

### 2.8. Effects of DON and Antibiotic-Induced Microbiota Depletion on Gut Flora Composition and Abundance

A total of 24 samples (six test samples in each group) were collected from the NovaSeq X Plus sequencing platform and filtered. The total number of data for each sample was 44,111,056 sequences, with a base number of 6,618,777,156 bp.

After quality control, the number of effective sequences was ≥98.76%, and the number of effective bases was ≥97.06%. The filtered data was then used for subsequent redundancy removal and analysis. Comparing the non-redundant gene set with the NR database, a LCA algorithm was used to preserve the biological significance to the maximum extent possible. A total of 5 domains, 13 boundaries, 137 phyla, 284 classes, 611 orders, 1363 families, 3686 genera and 12,982 species were annotated. The abundance statistics of annotated species at the phylum level are shown in Table 1. Among all species, *Verrucomicrobota* has the highest proportion at 32.90%, followed by *Bacteroideta* at 31.72% and *Bacillota* at 24.91%. These three phyla together account for 89.53% of the total abundance.

**Figure 3:**
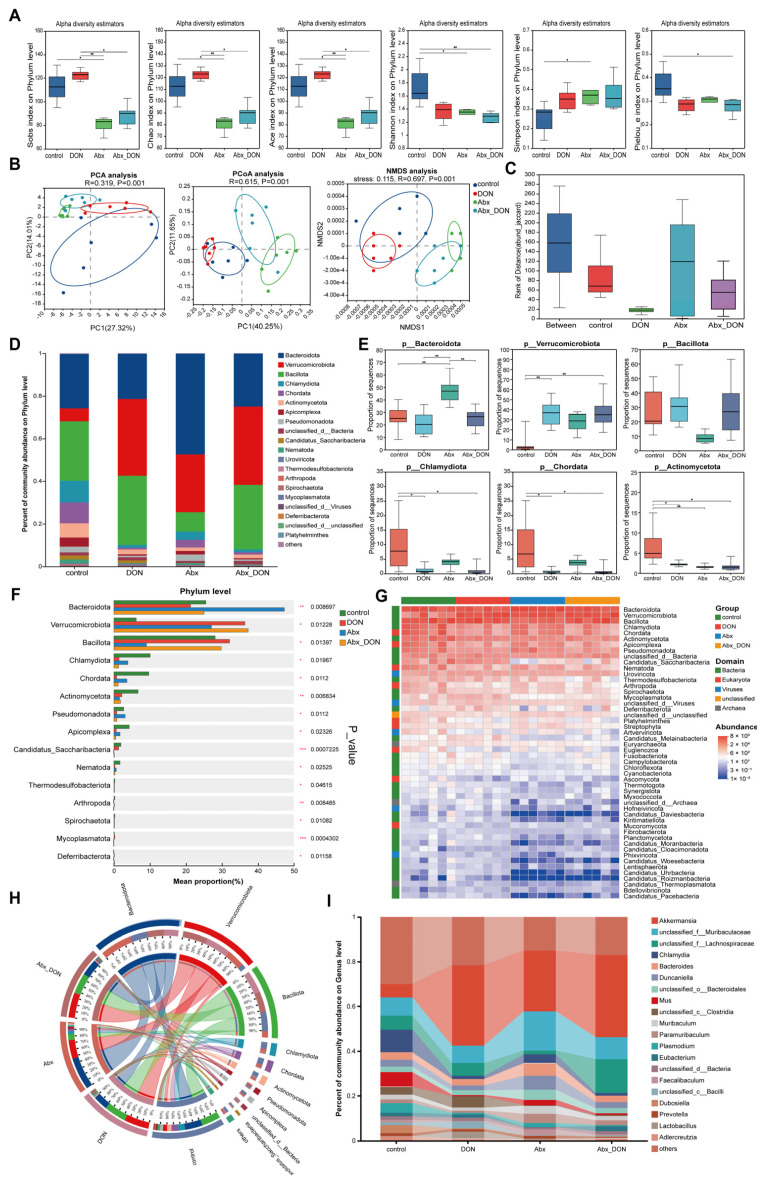
Effects of DON on intestinal flora species of mice during the experiment. (**A**) Alpha diversity index bar graph on Phylum level; (**B**) Principal component analysis on Phylum level, principal co-ordinates analysis on Phylum level and non-metric multidimensional scaling on Phylum level; (**C**) Analysis of similarities on Genus level; (**D**) on Phylum level; (**E**) The microorganisms with an abundance greater than 5.00% were analyzed in the box pattern; (**F**) Kruskal–Wallis H test bar plot on Phylum level; (**G**) Heatmap analysis on Phylum level; (**H**) Circos map of samples and species on Phylum level; and (**I**) Percent of community abundance on Genus level (n = 6). * 0.01 < *p* ≤ 0.05, ** 0.001 < *p* ≤ 0.01.

Annotation on the alpha diversity of the phylum level was conducted through mothur analysis, and the results are shown in Figure 3A. The indices of community richness were sobs, chao and ace, the indices of community evenness were Pieloue, simpsoneven and shannoneven, the community diversity index was Shannon and Simpson, whereas the index of community coverage was coverage. Each index reference can be found at: http://www.mothur.org/wiki/Calculators (accessed on 1 January 2019). The Sobs = Chao = Ace of each group of samples was measured, and the results showed that the Sobs = Chao = Ace of the DON group was significantly higher than the Abx DON group (*p* < 0.05). In addition, the control group had higher levels of Pielou-e compared to the other groups; however, the levels were only significantly higher than the Abx DON group (*p* < 0.05). The Shannon values in the control group were higher than the values in the other groups; however, they were significantly higher compared to the Abx DON group (*p* < 0.01). In contrast, the Simpson values in the control group were lower than those in the other groups, with a coverage = 1 for each group.

PCA, PCoA and NMDS analyses were conducted on the microbial community composition of each group at the phylum level based on the NR database. The results are shown in Figure 3B. The community composition between different groups was separated from each other (in each figure), and the Anosim test showed significant differences between groups (*p* < 0.05). ANOSIM analysis was also performed on microorganisms in each group at the genus level; and Figure 3C shows the results, that the differences between groups were significantly greater than the differences within groups (R = 0.62392, *p* = 0.001).

The bar plot of gut microbiota composition between different groups at the phylum level is shown in Figure 3D, with a total abundance of 100% for each group of samples. The main microbial communities that make up the colon microbiota in each group of mice were Bacteroidetes (21.27~47.28%), *Verrucomicrons* (6.19~37.19%) and Firmicutes (9.05~32.11%). The sum of these three phyla accounts for 59.81~91.92% of the total microbiota. Box plot analysis was performed on microorganisms with a richness greater than 5.00% (Figure 3E), as well as on the top 6 microorganisms with abundance. Among them, the phylum Bacteroidetes was the most abundant in the Abx group compared to control, DON and Abx DON groups (*p* < 0.01). The phylum *Verrucomicobacteria* had the lowest abundance in the control group compared to the DON and Abx DON groups (*p* < 0.01). The phylum Firmicutes had the lowest abundance in the Abx group compared to other groups; however, there was no significant difference when compared to other groups (*p* > 0.05). In addition, the control group had a higher abundance of Chlamydia (10.00%) compared to the other groups; however it was significantly higher than only the DON and Abx DON groups (*p* < 0.05). For the phylum Chordata, the control group had an abundance expression of 9.63% and showed similar group change trends with those observed in Chlamydia. The abundance of Actinobacteria in the control group (6.68%) was significantly higher compared to the other groups (*p* < 0.05). The box plot test performed for the abundance of other microbial phyla were less than 5.00%.

From Figure 3F, we observe that, compared with the control group, the DON group showed an increase in the richness of *Verrucomicrobia*, whereas the richness of other microorganisms decreased. In addition, compared with the Abx group, the Abx DON group showed an increase in the richness of *Verrucomicrons* and Firmicutes, while the richness of other microorganisms decreased. The DON and Abx DON groups showed similar trends in microbial richness changes.

The gut microorganism composition in the genus level is shown in Figure 3I. The results indicate that, after DON treatment, the abundance of *Akkermansia* increased whereas the abundance of *Muribaculaceae* decreased, and these changes were more pronounced in the presence of microbial communities.

**Figure 4:**
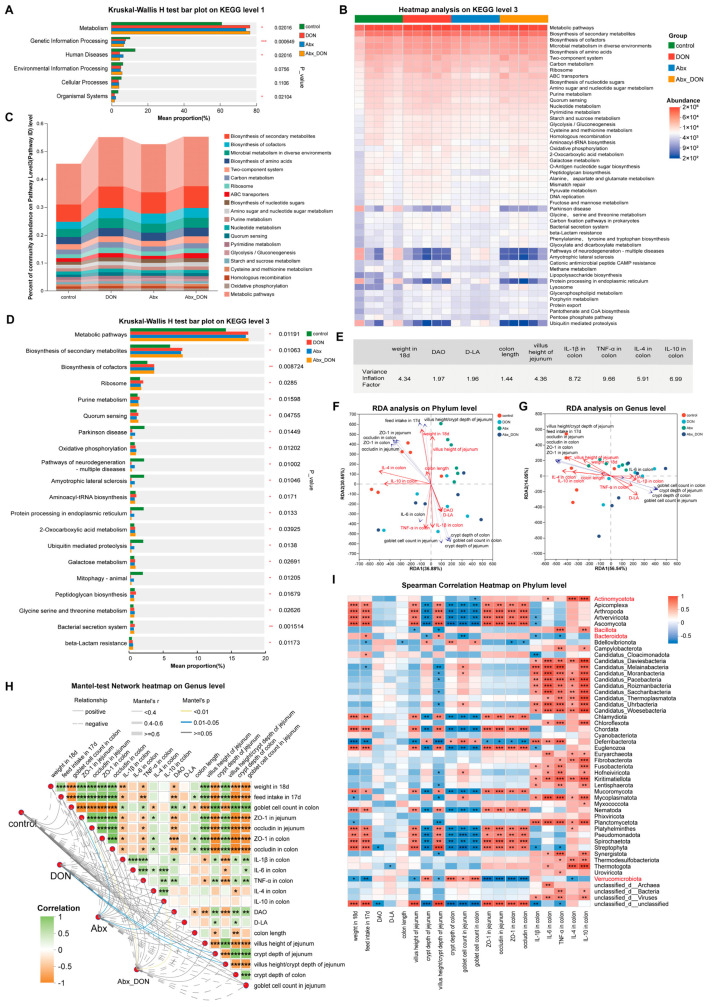
Effects of DON on KEGG pathway and joint analysis of species and pathological and inflammatory indicators. (**A**) Kruskal–Wallis H test bar plot on KEGG level 1; (**B**) Heatmap analysis on KEGG level 3, and each column represents a sample, the Y axis is the top 50 abundant channels and the abundance decreases from top to bottom; (**C**) Percent of community abundance on Pathway Level 3 (Pathway ID); (**D**) Kruskal–Wallis H test bar plot on KEGG level 3; (**E**) Variance inflation factor analysis results; (**F**,**G**) The CCA scatter plot of the sample distribution at the level of genus shows the distribution of samples and where the points represent the samples, and the different colors of the points represent samples from different groups. The distance between the points represents the similarity and difference in functional composition between the samples, and the pathological and inflammatory indicators are represented by arrows. The length of the arrow line represents the degree of correlation between the species distribution and the pathological and inflammatory indicators, with longer lines indicating a stronger correlation and shorter lines indicating a weaker correlation. The angle between the pathological and inflammatory indicators represents the positive or negative correlation between the pathological and inflammatory indicators (acute angle: positive correlation; obtuse angle: negative correlation; right angle: no correlation). (**H**) Mantel Test heat map, where the lines represent the correlation between the community and the pathological and inflammatory indicators, and the heat map represents the correlation between the pathological and inflammatory indicators. The thickness of the lines represents the correlation between the community and the pathological and inflammatory indicators, drawn using Mantel’s r (the absolute value of R). The solid and hollow lines represent positive and negative, respectively, indicating the correlation between the community and the pathological and inflammatory indicators. The different colors in the heat map represent the positive and negative correlations, with the depth of the color representing the size of the correlation, and the stars in the blocks representing significance. (**I**) The X and Y axes represent the indicators and species, respectively, with red representing positive correlation and blue representing negative correlation. On the Y-axis, the ones marked in red represent bacteria with genus abundance greater than 5.00%. * 0.01 < *p* ≤ 0.05, ** 0.001 < *p* ≤ 0.01, *** *p* ≤ 0.001 (n = 6).

In addition to changes in microbial composition, KEGG analysis (20230830, https://www.genome.jp/kegg (accessed on 9 February 2025)) was also performed. The microbiome function of mouse colon contents was analyzed, and a total of 11,960 KO numbers were identified. According to the KEGG database, at the first classification level, as Figure 4A shows, six functional gene clusters, including environmental information processing, cellular processes, human diseases, metabolism, organism systems, and genetic information processing, showed varying degrees of change, but they were mainly enriched in metabolism (*p* < 0.05). DON treatment increased the abundance of metabolism-related genes, and the increase was greater in the presence of microbial communities than in the absence of gut bacteria. At the three classification levels, as Figure 4B,C shows, the most abundant pathways were the metabolic pathway, biosynthesis of secondary metabolites, and microbial metabolites in diverse environments. As Figure 4D shows, Welch’s analysis was conducted on level 3 of each group. The results showed that the pathways that were significantly enriched included metabolic pathways, biosynthesis of secondary metabolites, biosynthesis of cofactors, ribosome, purine metabolism and quorum sensing. The abundance of these pathways in the Abx DON group was higher than that in the control group and Abx group (*p* < 0.05); however, the abundance in the DON group was higher compared to the Abx DON group. This suggests that DON may regulate the homeostasis of the intestinal environment by altering metabolic pathways.

Further, a cluster analysis was conducted on each sample in each group to identify the top 50 species in terms of richness (Figure 3G). High abundance and low abundance species were grouped together to reflect the similarities and differences in community composition among different groups/samples. As Figure 3G shows, the bacterial domain had the highest total abundance among the top 50 species in each group, and the community composition similarity at the phylum level was observed in the following order: control group ≠ Abx group ≠ DON group ≈ Abx DON group. To reflect on the visual correspondence between microorganisms at the phylum level in each group, richness analysis was conducted on the top 10 microorganisms with species richness using Circos plots (Figure 3H).

### 2.9. Joint Analysis on the Relationship Between Pathological and Inflammatory Indicators and Changes in Gut Microbiota

Changes in metabolic pathways affect pathological changes and inflammatory responses in the body, and changes in metabolites depend on changes in the abundance of the microbiota. Therefore, we jointly analyzed the relationship between the pathological and inflammatory indicators, including weight gain at 18d, feed intake at 17d, DAO, D-LA, colon length and crypt depth, villus height, crypt depth and villi height/crypt depth ratio of jejunum, goblet cell count in jejunum and colon, ZO-1 and occludin in jejunum and colon, and IL-1β, IL-6 in colon, TNF-α, IL-4, and IL-10 in colon with changes in gut microbiota species. We first used R language (version 3.3.1) and vegan (2.4.3) for variance inflation. For variance inflation factor (VIF) analysis, the larger the VIF value, the closer the multicollinearity relationship between the independent variables. It is generally believed that indicators with VIF values less than 10 are useful indicators. From this study, we observed that indicators with VIF values less than 10 included weight at 18d, DAO, D-LA, colon length, villus height of jejunum, IL-1B in colon, TNF-a in colon, 1L-4 in colon and 1L-10 in colon (Figure 4E). After the variance inflation test, we then performed the CCA analysis. The results at the phylum level are shown in Figure 4F (red arrows indicate factors with VIF < 10, and blue arrows indicate factors with VIF > 10). The results showed that the CCA1 axis and CCA2 axis functions accounted for 67.53% of the variables, among which weight at 18d, villus height of jejunum, and colon length were positively correlated, denoted as (I); IL-4 in colon and IL-10 in colon were positively correlated, denoted as (II); DAO, D-LA and IL-1βand TNF-α in colon are positively correlated, denoted as (III). However, we observed that (I) and (II) were negatively correlated with (III), indicating that the enzymes and compounds of intestinal damage in serum are positively related to inflammatory factors in the colon, and may have synergistic effects on the changes in the microbiota at the phylum level. The results at the genus level are shown in Figure 4G. The correlation of each indicator was roughly the same as that at the phylum level. The CCA1 axis and CCA2 axis functions accounted for 70.59% of the variables, but the positive correlation of (I) and (II) was stronger, and thus was donated as (IV). In addition, there was a stronger negative correlation between (IV) and (III) indicating that the changes caused by oral administration of DON, including the changes in pathological indicators, pro- and anti-inflammatory factors and tight junction proteins, which may have antagonistic or negative effects on microbiota at the genus level; thus, future research should focus on the changes in the pathways that regulate these indicators.

In order to further clarify the correlation between pathological and inflammatory indicators, the Mantel test statistical method was used to calculate the pathological and inflammatory indicators based on genus level. The results are shown in Figure 4H. The changes in animal feed intake and body weight caused by DON were positively correlated with morphological changes and tight junction protein changes (*p* < 0.01). The changes in DON group microbiota were positively correlated with crypt depth of jejunum and villus height/crypt depth of jejunum (*p* < 0.05). The changes in the Abx DON group’s microbiota were positively correlated with ZO-1 in jejunum and IL-10 in the colon (*p* < 0.01). In addition, based on the Spearman rank correlation coefficient, the correlation between phylum level microbial changes (based on relative abundance) and pathological/inflammatory indicators was studied. The clustering results are shown in Figure 4I. *Verrucomicrobiota* was negatively correlated with body feed intake, body weight and colon morphology measurements (*p* < 0.05) and positively correlated with tight junction proteins (*p* < 0.01), indicating that changes in *Verrucomicrobiota* abundance were associated with these significant indicators. It was interesting to observe that *Bacteroidota* and *Bacillota* exhibited completely opposite regulatory effects on various pathological and inflammatory indicators. This suggests that changes in pathological and inflammatory indicators are accompanied by changes in microbial abundance, further confirming the consistency between changes in pathological and inflammatory indicators and microbial abundance.

**Figure 5:**
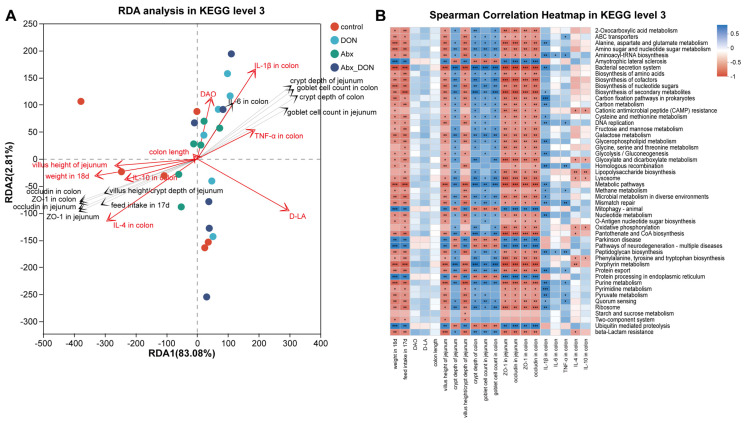
Correlation analysis between environmental factors and KEGG level 3. (**A**) RDA analysis in KEGG level 3; (**B**) Spearman correlation heatmap in KEGG level 3. * 0.01 < *p* ≤ 0.05, ** 0.001 < *p* ≤ 0.01, *** *p* ≤ 0.001 (n = 6).

Using the same calculation method, pathological and inflammatory indicators were jointly analyzed with KEGG level 3 (based on abundance) in each group. The results were shown in Figure 5A. At the KEGG level 3, the RDA1 and RDA2 axes accounted for 85.89% of the variables functionally. The indicators with VIF < 10 showed the same trend as those at the phylum and genus levels of microorganisms, except for the observed negative correlation between D-LA and DAO, which may be due to the different pathways that are involved by D-LA and DAO. Furthermore, we conducted a heatmap analysis on the relationship between pathological and inflammatory indicators and various pathways. As Figure 5B shows, among the top 50 pathways with total abundance at the classification level, most pathological and inflammatory indicators showed the same correlation trend, with weight at 18d, feed intake at 17d, villus height of jejunum, villus height/crypt depth ratio of jejunum, ZO-1 in jejunum, occludin in jejunum, ZO-1 in colon, occludin in colon and IL-1β in colon. We also observed a positive correlation between the pathological and inflammatory indicators in regulating metabolic pathways (*p* < 0.05), except for crypt depth of jejunum, crypt depth of colon and goblet cell count in jejunum and colon, which were negatively correlated in regulating metabolic pathways (*p* < 0.05).

## 3. Discussion

DON intake has negative effects on animal health, with the common clinical symptoms being reduced feed intake and weight loss [8]. In this experiment, mice with/without gut microbiota clearance were orally administered with 5 mg/kg BW DON for 14 days. The results showed that the feed intake and weight gain of the mice group with microbiota were worse compared to the mice with microbiota clearance (*p* < 0.05), which is consistent with the trend of Liao et al. [4], where 1 mg/kg BW/day gavage were administered to male C57BL/6j mice for 4 weeks. In addition, the current study also confirmed the dose dependent inverse relationship between that DON concentration and food intake and body weight [13]. Many studies have confirmed that intestinal microbes are related to host nutritional status, and as the homeostasis of intestinal flora are altered, absorption of intestinal nutrients are affected, which further leads to malnutrition of the body [14,15]. However, the results from our joint analysis of pathological and inflammatory indicators showed that weight in 18d is significantly correlated with microbial flora changes at gate level. Therefore, we speculated that the change of intestinal flora abundance may be one important reasons for the weight loss of mice.

The negative effects of DON on feed intake and body weight is related to the impact of DON on the digestive system [16]. The intestine is an important organ responsible for food intake, digestion, energy and nutrient absorption, immune response and excretion in humans and other animals [17]. The tissue structure of the small intestine is characterized by villi and crypts. DON cause toxicity to the intestine by altering its structure, disrupting the epithelial barrier, damaging mucosal immunity, and activating oxidative stress and gut dysbiosis [18,19]. However, whether the presence of microbiota enhances or reduces the activity of DON has not been fully investigated. Therefore, to investigate whether microbiota enhances or reduces the intestinal damage activity of DON, we administered DON to mice with or without gut microbiota clearance and evaluated the colon, length, histopathology, crypt depth and villi heights after the treatment period. We observed that the colon length of the mice without microbiota clearance was shorter compared to the mice with microbiota clearance, suggesting that the presence of microbiota may aggravate DON damage. The results from the pathological analysis revealed that the mice group with no gut microbiota clearance showed severe pathological changes in the mouse jejunum and colon, characterized by necrosis of epithelial cells and an increase in inflammatory cells after DON administration compared to mice with microbiota clearance. This result was consistent with the study by Hou et al. [20], which showed that DON administration caused pathological changes like inflammation in chicken.

Villi mainly play an absorptive role, and an increase in length enhances absorptive function, while a decrease in crypts mainly plays a secretory role [21]. The depth of the crypt reflects the maturation rate of intestinal epithelial cells [22]. The deeper the crypt, the lower the maturation rate of intestinal epithelial cells, and vice versa [23,24]. The ratio of villi height to crypt depth comprehensively reflects the health status of the intestine, and a decrease in the ratio indicates intestinal damage [25]. For the current study, we observed that the jejunal villi height of the mice group without microbiota clearance was significantly shortened compared to the group with microbiota clearance. In addition, the crypt depth and villi height/crypt depth ratio was deepened and decreased respectively in the group without microbiota clearance compared to the group with microbiota clearance. This was consistent with the study by Yang et al. [26]., which observed shortening of villi heights, deepening of crypt depth and decrease in villi height: crypt depth ratio in rabbits exposed to DON.

The goblet cells are known to be involved in the production of mucoprotein and the secretion of some factors that help in the repair of epithelial cells [27,28]. Previous studies have reported that DON at high doses can increase the number of goblet cells in villi and crypts both in vitro and in vivo [11,21]. The above observation was consistent with the results of our current study, in which we observed an increase in the number of goblet cells in mice without microbiota clearance compared to mice with microbiota clearance.

DAO and D-lactic acid are important chemical biomarkers used in measuring the integrity of the intestine [21]. These biomarkers are released into the bloodstream when the intestine is permeable as a result of damage. DAO catalyzes the oxidation of deamine, putrescine and histamine [29], whereas the D-LA is the product of anaerobic metabolism of glucose in the body. Increased DAO and D-LA levels are associated with diseases which induce severe intestinal injury [30]. Our results showed an increase in the levels of DAO and D-LA in the mice group without microbiota clearance compared to the mice group with microbiota clearance after DON administration; however, this was not significant.

The tight junction proteins serve as an important marker for determining permeability of the intestine. The reduction of these tight junctions has been associated with leaky gut [31]. Therefore, we evaluated the levels of occludin and ZO-1 in jejunum via immunofluorescence and RT-qPCR method. The results showed that the protein levels of ZO-1 and occludin were lower in the mice group without microbiota clearance compared to the mice group with microbiota clearance. This study was consistent with the study by Mi et al. [32], who reported that DON can reduce the expression of tight junction proteins (claudin and occludin in the mice colon). Studies have found that Firmicutes, Bacteroidetes, Actinobacteria and *Verrucomicrobia* are the most important bacterial groups in the intestinal microbiome of healthy animals, accounting for more than 90% of the total bacterial count [33]. These bacteria can produce bacteriocins and proteins that inhibit the growth of other specific harmful bacteria [34]. In addition, they produce amino acid metabolites through carbohydrate metabolism pathways [35], such as short-chain fatty acids (SCFAs) (such as propionate and butyrate) [36]. Although there are few metabolic changes caused by changes in the abundance of intestinal microorganisms under DON exposure, an increase in the metabolism of amino acids by intestinal microbiota was observed. It has been reported that increased amino acid metabolism may be harmful because it forms biogenic amines and procarcinogenic compounds [37], provides energy to intestinal epithelial cells, and helps improve epithelial function and promote upregulation of tight junctions (TJs) [38]. Studies have also shown that butyrate can prevent local inflammation and improve intestinal barrier permeability by restructuring TJs [39] and upregulating ZO-1 and occludin [40,41], while propionate and acetate can affect appetite and energy metabolism by regulating hormone levels and receptor activity. Our study found that DON can reduce the abundance of *Bacteroidota* and *Verrucomicrobiota*, and the reduction in the abundance of these flora reduces the ability to inhibit harmful flora. Our joint analysis of pathological and inflammatory factors showed that the expression levels of tight junction proteins were positively correlated with each other and were significantly affected by the changes in flora abundance. We speculated that DON ingestion caused changes in the abundance of flora in the colon of mice and affected the carbohydrate metabolic pathway, resulting in a decrease in the production of short-chain fatty acids by these flora, thus decreased the ability to produce TJs and exacerbating the destruction of the intestinal epithelial barrier. In contrast, due to the reduction of intestinal flora, the changes in flora abundance in mice that received gut microbiota clearance were much smaller than those in mice that did not received gut microbiota clearance, which further confirmed that the presence of intestinal flora may be a key factor in the exertion of intestinal toxicity induced by DON.

High expression of pro-inflammation cytokines, such as IL-1β and TNF-α, encourage intestinal epithelial cells damage via autocrine/paracrine action [35]. DON has been reported to cause damage to the intestine via activating inflammatory markers. Many researchers have proven that DON alters cytokines production and immune function [42,43]. In this study, we observed that the levels of pro-inflammatory cytokines (IL-1β, IL-6 and TNF-α) were significantly increased in the mice group without microbiota clearance compared to the mice group with microbiota clearance after DON administration. The study by Wu et al. [44] similarly showed an increase in the levels of inflammation cytokine proteins (IL-6, IL-8, and TNF-α) and mRNA expression of interleukin-6 in the jejunum and ileum of piglets after administration of DON. Studies have found that the occurrence of intestinal inflammation causes pro-inflammatory cytokines to migrate to the mucosal layer and then leak into the lumen [45]. The transport of these cytokines leads to the opening of tight junctions, resulting in intestinal leakage. After intestinal leakage, lipopolysaccharide, the main component of the bacterial outer membrane, enters the blood circulation and triggers a more severe inflammatory response. This finding is consistent with the results of our joint analysis of pathological and inflammatory factors. The reduction of tight junction proteins is positively correlated with the reduction of anti-inflammatory factors and negatively correlated with pro-inflammatory factors, and both are significantly regulated by changes in the abundance of microbiota. To date, scientists have still not clearly understood the impact of the presence of microbiota on the intestinal mechanical barrier under mycotoxin exposure, but through the comparison of microbiota removal experiments, we found that DON can promote inflammation by changing the abundance of microbiota in the intestine, destroying the structure of intestinal epithelial cells and reducing the synthesis of tight junction proteins. Therefore, the role of microbiota will become a new idea for exploring DON detoxification. Furthermore, gut dysbiosis alters the production of secondary metabolites which could contribute to the observed changes in cytokine levels. In the case of our study, we speculated that DON caused a shift in the production of pro-inflammatory cytokine related secondary metabolites thus the reason for increased level of IL-6, IL-8, and TNF-α observed.

Trpa1 is associated with decreased food intake as it plays a major role in the activation of exocytosis of CCK [46]. Studies have shown that the administration of DON increased in the expression levels of Trpa1 [47,48]. Similarly, we observed in this study that Trpa1 mRNA expression in the jejunum was significantly higher in the mice without microbiota clearance group compared to the mice with gut flora clearance. This confirms the reduction of feed intake observed in this studies.

DON administration disturbs the host’s gut microbiota homeostasis [49,50]. In the present study, we observed that DON exposure altered the relative abundance and composition of gut microbiota, which was similar to the results of a previous study by Liao et al. [4], who reported changes in the relative abundance of gut microbiota after administration of DON (1 mg kg^−1^ bw day^−1^) in mice for 4 weeks. DON causes an imbalance in the gut microbiota characterized by a decrease in *Bacteroidota* and *Actinomycota* abundance and an increase in *Verrucomicrobiota* and *Bacillota* abundance, resulting in changes in metabolic pathway abundance. We further observed that KEGG pathways, such as metabolic pathways, biosynthesis of secondary metabolites, biosynthesis of co factors, ribosomes, purine metabolism and quorum sensing, were significantly enriched in the gut microbiota after DON exposure, which supports previous arguments about gut toxicity [4,46]. We found that metabolic pathways were the most enriched pathway, followed by the biosynthesis of secondary metabolites pathway, which is related to the function and role of gut microbiota in metabolic processes, suggesting that DON intake activates pathways, such as carbohydrate metabolism, amino acid metabolism and lipid metabolism, and induced metabolic pathway activity with the participation of microbiota. An increase in amino acid metabolism amino acids can be harmful as it results in the formation of biogenic amines and precarcinogenic compounds that have negative effects on neurotransmission, immune regulation, cell proliferation and growth and antioxidant activity [37]. Therefore, although we did not separately detect changes in metabolites within the gut microbiota, previous studies have shown that the intake of DON can enhance the metabolic activity of gut microbiota towards amino acids, forming toxic metabolites such as ammonia, amines, phenols, and indole [41], which have adverse effects on gut cells and lead to diarrhea in pigs, thereby hindering growth performance [51]. Therefore, we can conclude that DON alters the gut microbiota and their metabolism, which in turn affects the gut function. Moreover, we observed from the study that there was a positive relationship between pathological and inflammatory indicators to changes in the gut microbiota composition which was consistent with previous studies that reported a strong association of intestinal damage parameters with changes in the relative abundance of gut microflora after DON exposure in previous studies [52]. Thus, we speculated that the changes in pathological and inflammatory indicators may be caused by changes in the intestinal flora which affects the KEGG pathway.

Furthermore, in this study, we observed that discrepant metabolic pathways may be involved in metabolism and biosynthesis of secondary metabolites after DON exposure, which also supports the previous demonstration of the toxicity in the intestine [4,46]. The reduction in the abundance of *Bacteroidota* and *Actinomycota*, along with the increase in *Verrucomicrobiota* and *Bacillota*, suggests that DON exposure disrupts the balance of the gut microbiota. This disruption may result in the alterations in microbial metabolism, as evidenced by the enrichment of the biosynthesis of secondary metabolites and purine metabolism pathway [53,54]. Secondary metabolites are compounds that are not essential for the basic life processes of microorganisms but play important roles in their interactions with the environment and other organisms, whereas purine metabolism is a critical process for the synthesis and degradation of purine nucleotides, which are essential for DNA and RNA synthesis, energy production, and cell signaling [54,55]. The changes in this pathway indicate that the gut microbiota may be responding to DON exposure by altering the production of these compounds and purine metabolites, which could have downstream effects on intestinal health. In addition, we observed significant association between pathological and inflammatory indicators with metabolic pathways. The observed changes in the biosynthesis of secondary metabolites pathway could contribute to the intestinal damage induced by DON as secondary metabolites produced by the gut microbiota may have pro-inflammatory or anti-inflammatory effects, which could influence the inflammatory response in the intestine. Additionally, changes in the production of secondary metabolites could affect the intestinal barrier function, further exacerbating the damage caused by DON. The increase in *Verrucomicrobiota* and *Bacillota*, a mucin-degrading bacterium, may indicate a compensatory mechanism to maintain intestinal barrier function in the face of DON toxicity. Therefore we speculated that the changes in pathological and inflammatory indicators may affect the KEGG pathways by influencing gut microbiota and their metabolites; however, there is the need for further studies to confirm this statement. Therefore, further exploration of how the microbiota can demonstrate DON toxicity damage through significantly correlated pathological and inflammatory indicators is our future research direction.

## 4. Materials and Methods

### 4.1. Experimental Animal

In total, 72 Specific pathogen free (SPF) male C57BL/6j mice (6–8 weeks) weighing 20 ± 2 g, were obtained from SiBeiFu (Beijing, China) and housed in specific pathogen-free facilities maintained at 22–24 °C with a 40–60% relative humidity and a 12-h light:dark cycle with no restrictions to food and water. The cages were disinfected daily.

### 4.2. DON Dosage Formulation and Determination

DON was purchased from Shanghai Yujing Technology Co., Ltd. (Shanghai, China) (purity ≥ 99.6%) and dissolved in deionized water to obtain a solution at a concentration of 1 mg mL^−1^. Each mouse received an appropriate solution by oral gavage 8:00 a.m. every morning to body weight (5 mg kg^−1^ bw day^−1^, equivalent to 0.55 mg kg^−1^ bw day^−1^ in human based on the Meeh–Rubner equation) for 14 days. The dosage and timing of DON were based on pre-experimental (unpublished data) and previous research results of our research group [56,57].

### 4.3. Antibiotics Treatment

Vancomycin and metronidazole were purchased from Shanghai Yuanye Biotechnology Co., Ltd. (Shanghai, China), while neomycin sulfate and ampicillin were purchased from Shanghai Yien Chemical Technology Co., Ltd. (Shanghai, China) Four antibiotics were used to create a sterile mouse model. The mice were orally administered following the procedure of Gong et al. [58]. Vancomycin 100 mg/kg B.W, neomycin sulfate 200 mg/kg B.W, metronidazole 200 mg/kg B.W, and ampicillin 200 mg/kg B.W were dissolved in 1× PBS solution and administered orally every morning at 8 a.m. for 5 days.

### 4.4. Experimental Design

**Figure 6:**
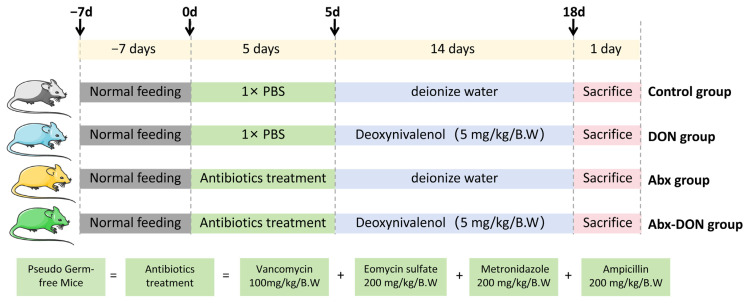
Experimental groups and their treatments.

After 7 days adaptation period, mice were randomly allocated into four groups with a total of 12 mice in each group. The control group received only 1× PBS + deionized water, the DON group received 1× PBS + DON solution, the Abx group received antibiotics + deionized water, and the Abx-DON group received antibiotics + DON solution. The summary information of the treatment groups is shown in Figure 6.

After the treatment period, mice in each group were fasted for 4 h and 12 mice were randomly selected from each group and sacrificed following euthanization with isoflurane (1.5% at a flow rate of 0.4–0.8 L/min). Blood was collected from the right eye using retroorbital blood collection method. After being transferred into an ep tube and left at room temperature for 30–60 min, the blood samples were then centrifuged at 4000 r/min for 10 min, 4 °C to obtained serum, which was stored at −20 °C until further analysis. In addition, intestinal tissue was then aseptically removed, weighed, and 2 cm in length of the jejunum tissue (in the middle segment of jejunum) and colon tissue (2–7 cm proximal to the anus) were cut for further analysis. The length of the colon was measured using a meter rule. The colon contents were aseptically collected 4 cm from the anus, and were quickly frozen in liquid nitrogen for further analysis (n = 6).

### 4.5. Clinical Observation

After 7 days of adaptive feeding, the experimental phase was initiated. During this period, the body weight and feed intake of each group of mice were measured and recorded at 8 a.m. every day. At the same time, the mental, hair, and fecal status of each group of mice were observed to see if there was any soft stool or diarrhea, as well as clinical symptoms of poisoning, which were recorded.

### 4.6. Enzyme-Linked Immunosorbent Assay Detection on DAO and D-LA in Mice

The concentrations of diamine oxidase (DAO) and D-Lactic acid (D-LA) were determined in the blood serum using commercial ELISA kits (Jingmei Biological Technology, Jiangsu, Yancheng, China) to assess the degrees of gut mucosal barrier damage according to the manufacturer’s instructions. The level of sensitivity of each kit was 1 μg/mL (DAO: pg/mL, D-LA: μg/L).

### 4.7. Pathology of Gut Tissue

#### 4.7.1. Hematoxylin-Eosin Staining

Mice jejunum and colons were fixed in 4% paraformaldehyde for 36 h, embedded in paraffin blocks, and cross-sectioned at 4 μm thickness using a conventional rotary slicer (Leica, RM2235, Nußloch, Germany). After deparaffinization, sections were successively stained with hematoxylin for 3 min and eosin for 5 min for morphological analysis. The 3DHISTECH (Budapest, Hungary) Pannoramic SCAN digital slice scanner and CaseViewer 2.3 software were used to observe and analyze the pathological changes in jejunum and colon. Pathology of jejunum and colon sections as well as villi height and crypt depth were measured using an Image Pro Plus 6.0 (Media Cybernetics, Bethesda, MD, USA).

#### 4.7.2. AB-PAS Staining and GCs Counting

The paraffin blocks were cut into 4 µm thick slices using a slicer. The slices were deparaffinized, hydrated, stained with Alizarin blue staining solution, and oxidized with 1% periodic acid oxidant. After staining with Schiff’s solution, the cell nucleus was stained with hematoxylin, differentiated with hydrochloric acid and alcohol, and subjected to routine dehydration, transparency, and sealing. 3DHISTECH (Budapest, Hungary) Pannoramic SCAN digital slice scanner was used to capture images, whereas CaseViewer software was used to observe the images. In addition, the Image Pro Plus 6.0 image analysis system was used to measure the tissue area under the field of view to determine the number of goblet cells per unit area (cells/μm^2^).

### 4.8. Immunofluorescence Detection of Tight Junction Proteins (TJs) Protein Localization and Expression Levels

The IF staining was conducted as previously described (Lei et al. [59]). The primary antibodies included ZO-1 (cat. no. #AF5145; 1:100; Affinity Biosciences, Cincinnati, OH, USA) and Occludin (cat. no. #DF7504; 1:200; Affinity Biosciences, Cincinnati, OH, USA). The Alex Fluor 488-labeled secondary antibodies (Invitrogen, Carlsbad, CA, USA) were used for the analysis. Images were obtained through the Fluorescence microscope (CX40, SOPTOP, Ningbo, China). The Image Pro Plus 6.0 image analysis system measures the optical density of each segment of the intestine, and the average optical density reflects the expression of ZO-1 and occludin proteins.

### 4.9. Western Blot Detection of DON Expression of Inflammatory Factors

RIPA lysis buffer (P0013B, Beyotime, Shanghai, China) supplemented with protease inhibitors was used to extract total protein from the mice jejunum and colon tissues. Proteins were transferred to nitrocellulose membranes after separation by 12% sodium dodecyl sulfate-PAGE (SDS-PAGE). The proteins were probed with primary antibodies against IL-1 β, IL-6, TNF-α, IL-4, and IL-10 GAPDH (abs137959, 1:10,000, Absin, Shanghai, China). Secondary anti-mouse/rabbit IgG, HRP-linked antibody (#7076, #7074, 1:10,000, Cell Signaling Technology, Danvers, MA, USA), and Lumigen ECL Ultra (Lumigen, Southfield, MI, USA) detection reagents were used to visualize the proteins.

### 4.10. RNA Extraction and Quantitative Real-Time PCR Analysis

Following the method by Cui et al. [21], the intestinal tissues stored in the −80 °C were subjected to RNA extraction. Specific primers were designed for *ZO-1*, *occludin*, *claudin-3*, *IL-1β*, *IL-6*, *TNF-α*, *IL-4*, *IL-10* and *Trpa1* genes using Oligo 7.0 software, and synthesized by Shanghai Biotech Co., Ltd. (Shanghai, China) Every intestinal segment was opened longitudinally, cleaned by PBS, snap frozen in liquid nitrogen, and grinded into powder with a mortar and pestle. The total RNA from each sample was extracted with an Animal Total RNA Isolation Kit (Sagon Biotech, Shanghai, China) following the manufacturer’s instructions. RNA concentrations were determined by absorbance at 260 nm in a UV spectrophotometer (Thermo Fisher Scientific, Waltham, MA, USA; OD260/280 ≈ 1.9–2.0). After 2 μg RNA was reverse transcribed into 20 μL cDNA using a PrimeScrip RT reagent kit (Takara, Tokyo, Japan), quantitative real-time PCR was performed by using a CFX96 PCR detection system (BioRad, Hercules, CA, USA) with a SYBR Premix ExTaq (Takara). The conditions of PCR were as follows: 95 °C for 5 min, then 40 cycles of 95 °C, 15 s for denaturation, 60 °C, 60 s for annealing at 70 °C and 25 s for extension. Each qRT-PCR reaction was performed with volumes of 15 µL containing 6.25 µL TB Green TM Premix (Takara), 0.3 µL forward and reverse primers, 1.5 µL cDNA, and 6.65 µL DNase/RNase-Free Deionized Water (Tiangen, Beijing, China). The gene primers used in this experiment are shown in Table 2. The relative expression levels were normalized by internal reference GAPDH through the 2^−ΔΔCt^ method.

### 4.11. Total DNA Extraction and Sequencing

The quality testing of DNA samples and library construction procedures were carried out in accordance with the standards of Shanghai Meiji Biomedical Technology Co., Ltd. (Shanghai, China). After extracting the total DNA from the collected colonic contents with the DNA extraction kit, NanoDrop2000 (Thermo, Wilmington, DE, USA) was used to detect the DNA purity, TBS-380 was used to detect the DNA concentration, and 1% agarose gel electrophoresis with voltage of 5 V/cm, and time 20 min was used to detect the DNA integrity. The Covaris M220 ultrasonic disruptor (Covaris, Woburn, MA, USA) was used to break DNA into fragments of approximately 400 bp and NEXTFLEX Rapid DNA Seq Kit (Revvity, Waltham, MA, USA) was used for the construction of the library. Furthermore, NovaSeq X Plus (Illumina, San Diego, CA, USA) sequencing platform was used for metagenomic sequencing (Shanghai Meiji Biomedical Technology Co., Ltd.).

### 4.12. Statistical Analysis

SPSS 25.0 statistical analysis software was used to perform normality tests on experimental data. For experimental data that conformed to normality, homogeneity of variance tests were performed. Two way ANOVA tests were used for experimental data that conformed to normality and homogeneity of variance. Independent sample *t*-tests were performed for each of the four “selected cases”, and the differences between the groups were shown in a bar chart. All experimental data are expressed as “Mean ± SD”, ns indicates no difference, “*” *p* < 0.05 indicates significant difference, and “**” *p* < 0.01 indicates extremely significant difference.

## 5. Conclusions

From the study, we conclude that gut microbiota presence aggravates the damaging activity of DON to the intestine. However, the mechanism by which gut microbiota enhance the toxicity of DON still needs further research. In addition, one major limitation of this study is the lack of a validation experiment to confirm the hypothesis by restoring the gut microbiota of mice with microbiota clearance with probiotics or microbiota transplant to observe whether the toxic activity of the DON would elevate. Furthermore, this study did not investigate how gut microbiota presence causes inflammatory responses and reduced gut integrity in the various intestinal tissues after DON administration, as well as the role of gut microbiota metabolites on DON associated gut toxicity. We plan to investigate this as our future experiment.

### Significance of the Study

Mycotoxins cause various health complications which could even lead to death. Over the past decades, the fight against the existence of mycotoxin in our food has been unwinnable as it is very difficult to get rid of these toxins. Thus, this raises a serious health threat to humans and animals who come into contact with these toxins every day. As the intestine is the first organ to interact with mycotoxins, various studies have studied mycotoxins’ effects on the intestinal function as well as intestinal microbiota. However, the involvement of the gut microbiota in the toxicity effect has not been fully studied. Therefore, for this to be investigated, we observed the degree of damage of the intestine in the presence or absence of the gut microbiota to give the scientific basis for further independent studies on the molecular mechanism of the gut microbiota in the degree of damage by various mycotoxins. The results from this study will serve as basic knowledge for further studies into the exact molecular mechanism and biology involved in gut microbiota aggravation of intestinal damage induced by DON, thus helping in the development of biological strategies that will equip the gut microbiota to help reduce the toxicity of mycotoxins after ingestion by both humans and livestock.

## Figures and Tables

**Table 1:** NR species annotation results (top 5 phyla).

Top	Domain	Phylum	Total_reads	Percent	Prevalence
1	d__Bacteria	p__*Verrucomicrobiota*	149,990,662	32.90%	1
2	d__Bacteria	p__*Bacteroidota*	144,624,306	31.72%	1
3	d__Bacteria	p__*Bacillota*	113,552,972	24.91%	1
4	d__Bacteria	p__*Actinomycetota*	14,673,708	3.22%	1
5	d__Bacteria	p__*Chlamydiota*	7,838,146	1.72%	1

Note: Total_reads is the total abundance of a species in all samples; percentage is the proportion of a species in the total abundance of all samples; prevalence is the frequency at which a species appears in a sample.

**Table 2:** Primers.

Gene Name	Primer	Sequence	Annealing Temp.
*IL-1β*	F: TGGTGTGTGACGTTCCCATT	16,176	60/60
	R: TGTCGTTGCTTGGTTCTCCT		
*IL-6*	F: TGTCGTTGCTTGGTTCTCCT	16,193	60/60
	R: TGCAAGTGCATCATCGTTGTTC		
*TNF-α*	F: TCTTCTCATTCCTGCTTGTGG	21,926	60/60
	R: ATGAGAGGGAGGCCATTTG		
*IL-4*	F: ACGGAGATGGATGTGCCAAAC	16,189	60/63
	R: AGCACCTTGGAAGCCCTACAGA		
*IL-10*	F: GCCAGAGCCACATGCTCCTA	16,153	61/61
	R: GCCAGAGCCACATGCTCCTA		
*ZO-1*	F: ACCAGATGTGGATTTACCCGTCA	21,872	61/60
	R: ACATCATTTCCACCAGCTAGTCG		
*Occludin*	F: GGCAAGCGATCATACCCAGA	18,260	60/60
	R: GCTGCCTGAAGTCATCCACA		
*Trpa1*	F: GGCAATGTGGAGCAATAGCG	277,328	60/60
	R: CCGGTCGATCTCAGCAATGT		
*claudin 3*	F: CCCTCATCGTGGTGTCCATC	12,739	60/60
	R: CGTCTCGTCTTGTACGCAGT		
*GAPDH*	F: CGACTTCAACAGCAACTCCCACTCTTCC	14,433	60/60
	R: TGGGTGGTCCAGGGTTTCTTACTCCTT		

## Data Availability

The data presented in this study are available on request from the corresponding authors.
